# Supplementary material for: Ginkgolide B Regulates CDDP Chemoresistance in Oral Cancer via the Platelet-Activating Factor Receptor Pathway
Source: Cancers (Basel). 2021 Dec 15;13(24):6299. doi: 10.3390/cancers13246299 (PMC8699349; doi:10.3390/cancers13246299)
Supplement: Supplementary file 1 [file cancers-13-06299-s001.zip › cancers-1502338-supplementary.pdf]

Supplementary Materials

Ginkgolide B Regulates CDDP Chemoresistance in Oral Cancer via the Platelet-Activating Factor Receptor Pathway

Kohei Kawasaki, Atsushi Kasamatsu, Toshiaki Ando, Tomoaki Saito, Takafumi Nobuchi, Ryunosuke Nozaki, Manabu Iyoda and Katsuhiro Uzawa

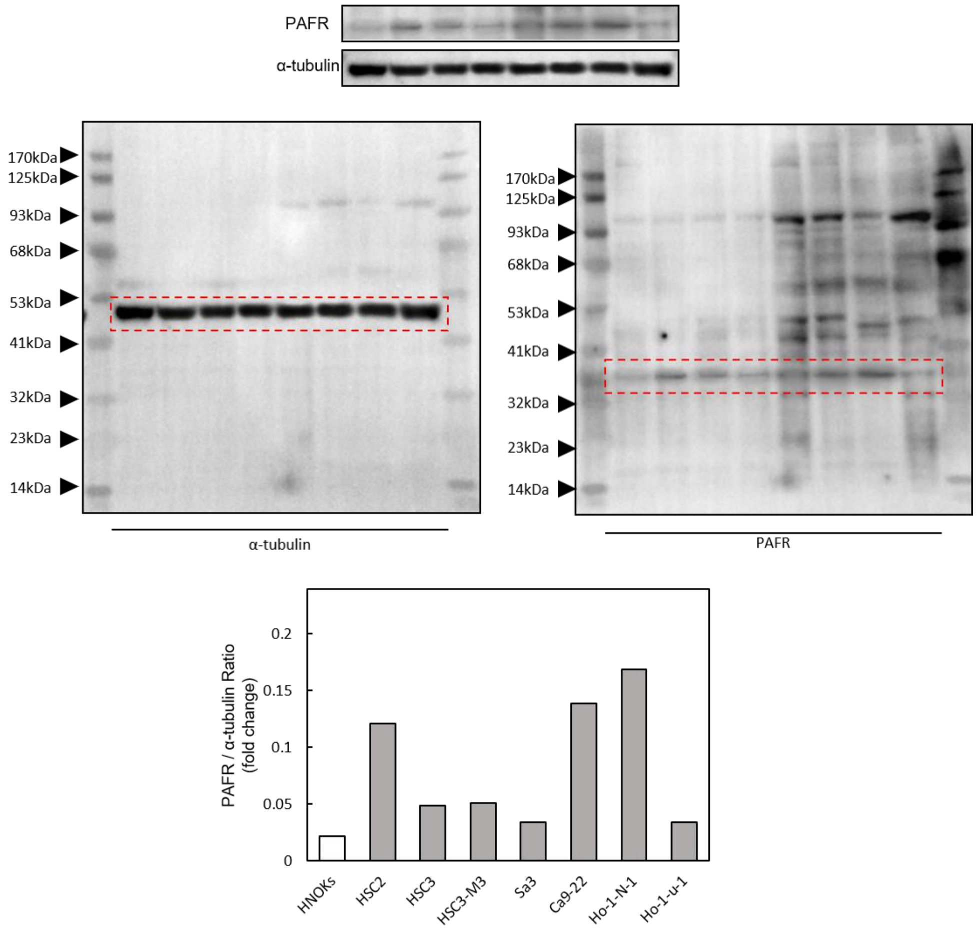

Figure S1. Full length blots of Figure 1B.

Table S1. Densitometry readings/intensity ratio of each band for blots of Figure 1B.

Cell Line	PAFR	α-tubulin	PAFR/α-tubulin
HNOKs	271,134.67	12,727,560.00	0.021302958
HSC2	802,338.23	6,633,935.00	0.120944542
HSC3	524,384.61	10,752,920.00	0.048766717
HSC3-M3	441,746.72	8,643,179.00	0.051109287
Sa3	296,266.51	8,658,495.00	0.03421686
Ca9-22	1,178,130.69	8,473,819.00	0.139031845
Ho-1-N-1	1,449,447.00	8,579,264.00	0.168947709
Ho-1-u-1	282,764.59	8,408,593.00	0.03362805

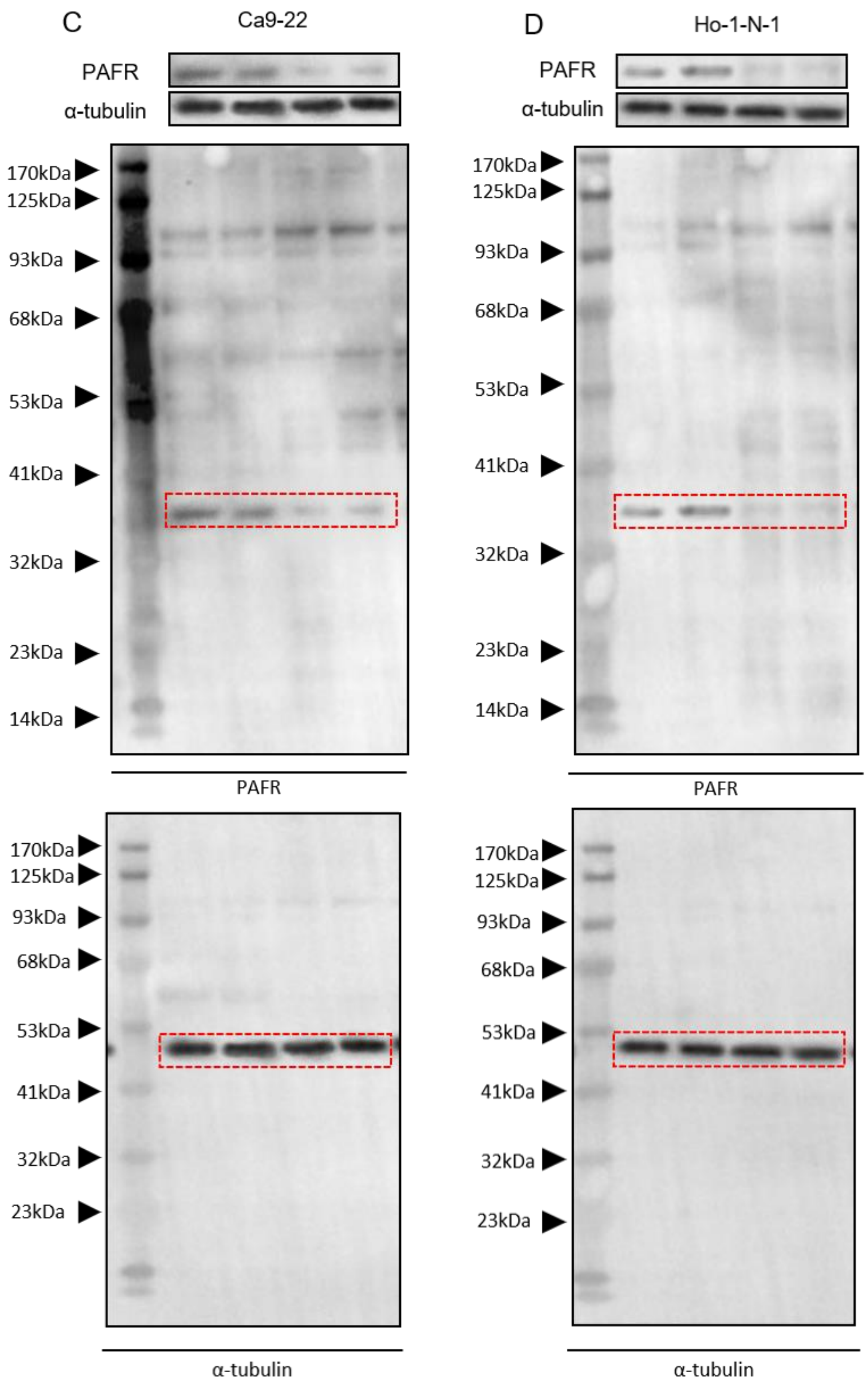

Figure S2. Full length blots of Figure 3C,D.

Table S2. Densitometry readings/intensity ratio of each band for blots of Figure 3C,D.

**Cell Line**	**Treatment**	**PAFR**	**α-tubulin**
Ca9-22	mock	545,059.15	3,299,895.03
	control	546,936.03	3,601,183.13
	si*PAFR*-1	150,095.59	3,751,964.06
	si*PAFR*-2	299,206.89	3,588,765.82
		**PAFR**	**α-tubulin**
Ho-1-N-1	mock	642,385.05	3,741,251.51
	control	678,928.18	4,160,239.00
	si*PAFR*-1	218,990.13	3,851,011.70
	siPAFR-2	251,711.88	3,876,946.08
		**PAFR/α-tubulin**	
Ca9-22	mock	0.165174693	
	control	0.151876761	
	si*PAFR*-1	0.040004538	
	si*PAFR*-2	0.0833732	
		**PAFR/α-tubulin**	
Ho-1-N-1	mock	0.171703252	
	control	0.163194514	
	si*PAFR*-1	0.05686561	
	si*PAFR*-2	0.064925298	

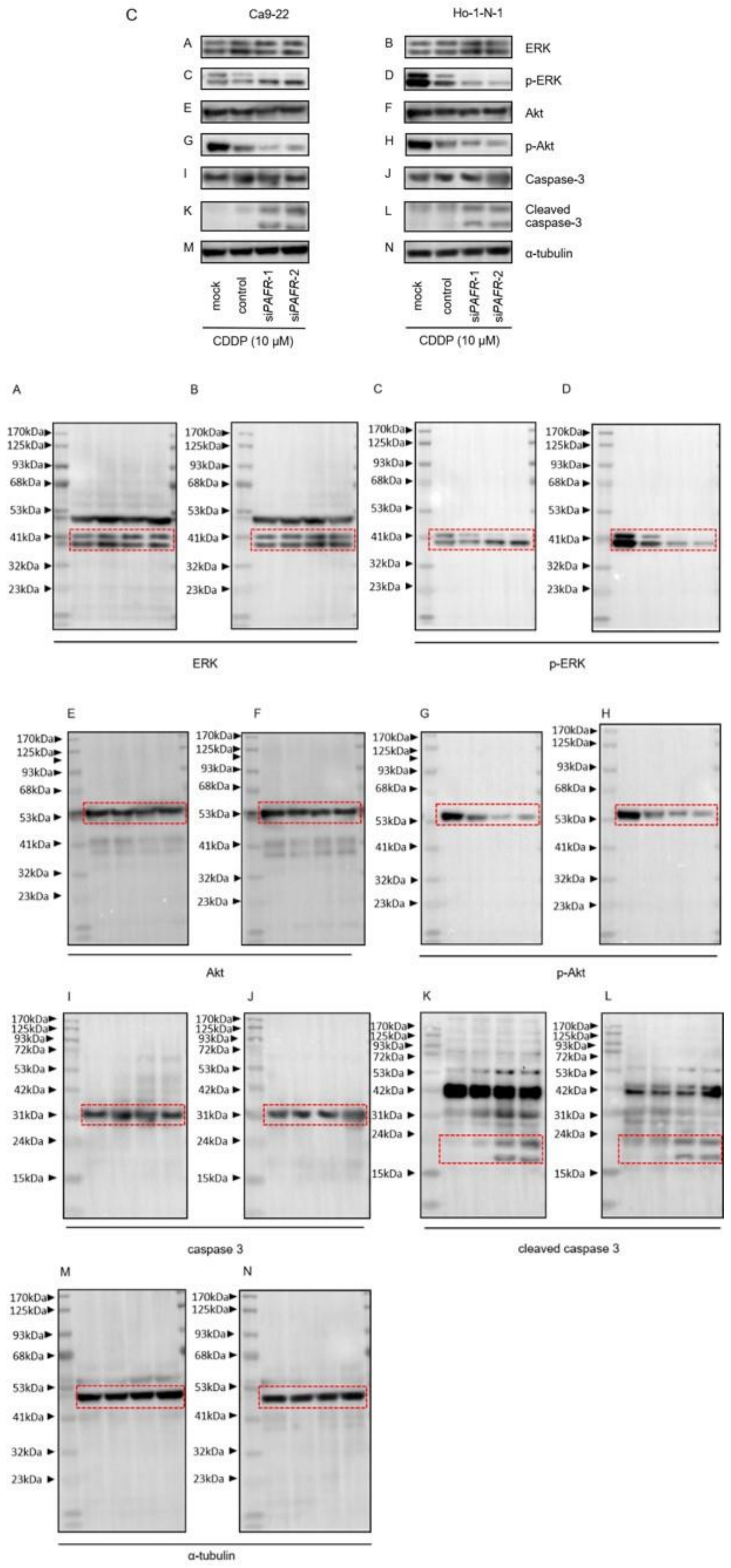

Figure S3. Full length blots of Figure 5C.

Table S3. Densitometry readings/intensity ratio of each band for blots of Figure 5C.

**Cell Line**	**Treatment**	**ERK**	**p-ERK**	**Akt**	**p-Akt**	**Caspase 3**	**Cleaved Caspase 3**	**α-tubulin**
Ca9-22	mock	1,356,531.46	3,215,954.88	2,764,251.84	3,297,971.96	3,592,708.68	61,272.72	2,566,574.30
	control	2,309,543.50	2,484,591.27	2,900,993.82	2,406,840.00	4,544,902.74	105,181.64	2,597,377.21
	si*PAFR*-1	1,766,964.96	1,548,984.54	2,919,050.69	418,752.69	4,158,652.76	337,876.54	2,081,260.50
	si*PAFR*-2	1,637,630.22	1,889,635.51	2,512,724.06	1,241,790.82	4,444,442.33	461,972.14	3,030,881.43
		ERK	p-ERK	Akt	p-Akt	Caspase 3	Cleaved Caspase 3	α-tubulin
Ho-1-N1	mock	2,234,600.88	10,149,673.75	3,301,442.54	6,006,537.31	5,295,274.43	71,106.42	2,996,538.37
	control	1,794,416.00	2,271,717.06	3,678,785.98	2,085,885.73	4,987,485.44	87,045.59	2,529,115.75
	si*PAFR*-1	2,893,110.21	1,362,577.56	3,500,105.76	632,686.00	3,035,688.51	282,056.80	2,400,054.21
	si*PAFR*-2	1,643,520.63	1,046,864.88	3,703,172.00	47,883.70	4,441,634.19	322,254.24	2,987,692.00

		ERK/ α-tubulin	p-ERK/ α-tubulin	Akt/ α-tubulin	p-Akt/ α-tubulin	Caspase 3/ α-tubulin	Cleaved Caspase 3/ α-tubulin
Ca9-22	mock	0.528537771	1.253014526	1.077019995	1.284970383	1.399807003	0.023873347
	control	0.889182939	0.956576989	1.116893537	0.926642457	1.749804658	0.040495327
	si*PAFR*-1	0.84898789	0.744253081	1.402539802	0.201201479	1.998141396	0.162342263
	si*PAFR*-2	0.540314842	0.623460717	0.829040699	0.409712768	1.466386077	0.152421713
		ERK/ α-tubulin	p-ERK/ α-tubulin	Akt/ α-tubulin	p-Akt/ α-tubulin	Caspase 3/ α-tubulin	Cleaved Caspase 3/ α-tubulin
Ho-1-N-1	mock	0.745727437	3.387132917	1.101752133	2.00449204	1.767130527	0.023729521
	control	0.709503312	0.898225817	1.454573987	0.824749018	1.972027354	0.034417401
	si*PAFR*-1	1.20543536	0.567727826	1.45834446	0.263613212	1.264841643	0.117521012
	si*PAFR*-2	0.550097075	0.350392504	1.239475823	0.016026987	1.486643935	0.107860596

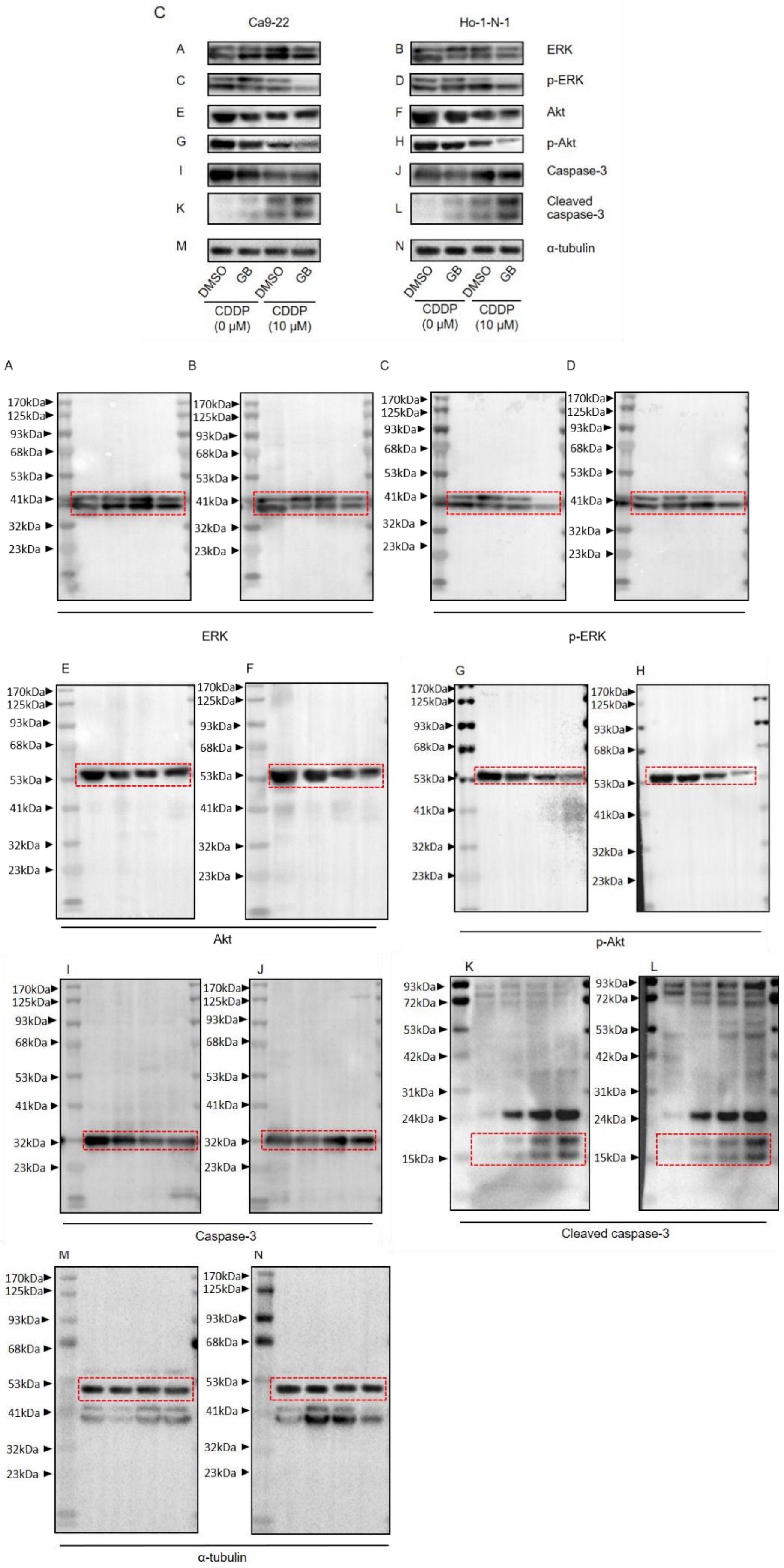

Figure S4. Full length blots of Figure 8C.

Table S4. Densitometry readings/intensity ratio of each band for blots of Figure 8C.

Cell Line	Treatment	ERK	p-ERK	Akt	p-Akt	Caspase 3	Cleaved Caspase 3	α-tubulin
Ca9-22	DMSO	2,881,850.75	5,823,743.33	1,145,574.69	4,615,888.30	10,546,622.03	3,058,261.06	3,674,654.54
	GB	2,862,435.37	5,058,438.71	1,096,822.20	4,289,872.47	9,128,778.00	4,276,263.37	3,421,368.29
	CDDP	3,584,508.43	1,897,166.46	991,284.31	2,238,238.10	6,782,509.62	8,043,499.69	3,542,937.32
	GB+CDDP	2,778,551.69	597,242.70	1,108,217.33	1,359,071.07	7,798,705.70	16,284,861.19	3,814,041.25
		**ERK**	**p-ERK**	**Akt**	**p-Akt**	**Caspase 3**	**Cleaved Caspase 3**	**α-tubulin**
Ho-1-N1	DMSO	3,328,744.73	3,743,208.53	2,945,861.16	4,164,088.21	7,255,405.76	3,044,909.50	3,431,678.97
	GB	2,466,626.95	4,209,819.41	2,565,154.42	4,061,629.70	6,311,717.40	4,314,978.71	4,209,041.00
	CDDP	3,567,043.42	2,627,174.15	1,825,434.50	2,146,610.81	8,162,529.88	8,358,677.27	3,291,992.57
	GB+CDDP	3,223,696.17	920,484.53	1,965,574.33	1,007,867.09	7,255,049.44	16,807,416.81	3,595,028.84
		**ERK/ α-tubulin**	**p-ERK/ α-tubulin**	**Akt/ α-tubulin**	**p-Akt/ α-tubulin**	**Caspase 3/ α-tubulin**	**Cleaved Caspase 3/ α-tubulin**	
Ca9-22	DMSO	0.784250797	1.584841042	0.311750309	1.256142026	2.870098921	0.832258098	
	GB	0.836634682	1.478484127	0.320579986	1.253846893	2.668165841	1.249869245	
	CDDP	1.011733516	0.535478415	0.279791659	0.631746429	1.914374714	2.270291082	
	GB+CDDP	0.728505936	0.156590519	0.290562492	0.356333587	2.044735541	4.269712867	
		**ERK/ α-tubulin**	**p-ERK/ α-tubulin**	**Akt/ α-tubulin**	**p-Akt/ α-tubulin**	**Caspase 3/ α-tubulin**	**Cleaved Caspase 3/ α-tubulin**	
Ho-1-N-1	DMSO	0.9700047	1.090780508	0.858431452	1.213425858	2.11424373	0.887294245	
	GB	0.58603063	1.000184938	0.609439162	0.964977295	1.49956187	1.025168916	
	CDDP	1.08355148	0.798049842	0.554507479	0.652070123	2.47951042	2.539093519	
	GB+CDDP	0.896709404	0.256043712	0.546747861	0.280350185	2.01807823	4.675182522	

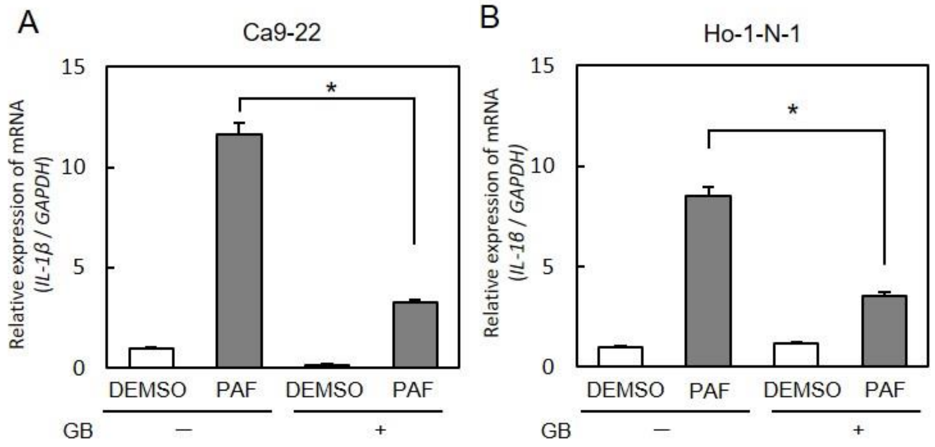

Figure S5. Confirmation of GB inhibition. To confirm the GB inhibitory effect, we investigated the expression level of IL-1β, an inflammatory cytokine induced by PAF. OSCC cells were treated with PAF (50 nM) and/or GB (200 μM), and the expression level of IL-1β mRNA was quantified by qRT-PCR analysis. Expression of IL-1β mRNA induced by PAF was significantly down-regulated after treatment with GB compared to the cells treated with PAF alone (Ca9-22 (**A**) and Ho-1-N-1 (**B**); Unpaired *t*-test; *, $p < 0.001$). GB, ginkgolide B; PAF, platelet-activating factor; OSCC, oral squamous cell carcinoma.

© 2021 by the authors. Licensee MDPI, Basel, Switzerland. This article is an open access article distributed under the terms and conditions of the Creative Commons Attribution (CC BY) license (http://creativecommons.org/licenses/by/4.0/).
